# Progress on Seasonal and Pandemic Influenza Vaccines

**DOI:** 10.3390/vaccines9101068

**Published:** 2021-09-24

**Authors:** Claudia Maria Trombetta, Emanuele Montomoli

**Affiliations:** Department of Molecular and Developmental Medicine, University of Siena, 53100 Siena, Italy; emanuele.montomoli@unisi.it

Influenza is a vaccine-preventable disease and vaccination is the most effective way of controlling seasonal influenza infections and preventing possible pandemic events. Due to the evolving nature of influenza viruses, the composition of vaccines needs to be updated annually in order to include the seasonal viruses predicted to circulate during the next influenza season [1].

To date, three different classes of influenza vaccines have been licensed: inactivated, live attenuated, and recombinant haemagglutinin vaccines. The latter have recently been licensed and contain haemagglutinin only. The inactivated vaccines are the most widely used as subunit or split viruses. They are still mainly produced in 9–11-day-old pathogen-free eggs, an old fashioned but well-established process that presents some drawbacks. The live attenuated influenza vaccines (LAIVs) are administered intranasally to healthy subjects of different ages depending on the country regulations. The intranasal administration mimics the natural pathway of infection inducing a broad humoral and cellular response in addition to a mucosal IgA response in the upper respiratory tract [2,3].

This Special Issue of *Vaccines*, entitled *Progress on Seasonal and Pandemic Influenza Vaccines*, covers the latest findings on influenza vaccines, including different types of vaccines and new technologies and platforms under development for next-generation vaccines.

The first published paper [4] describes a competitive ELISA method that is able to detect specific anti-stalk serum IgG antibodies against conserved epitopes among group 1 and group 2 influenza A viruses. This potential method, used in parallel with traditional immunological assays, could be of relevance for the evaluation of the immune response of next-generation influenza vaccines, such as the universal vaccine. 

Domnich et al. [5] provide a well-organized overview of 1164 registered clinical trials aimed at describing and analyzing the use of the available immunological assays for quantifying the immunogenicity of licensed influenza vaccines and candidates. Most of the studies evaluated the humoral response using a single assay only, usually the haemagglutination inhibition assay.

As we learned from the COVID-19 pandemic, it is difficult to predict which influenza virus may cause the next pandemic; however, we need to be aware that influenza A viruses could cause a worldwide outbreak of influenza to which people lack protective immune responses. As pigs have both human and avian receptors, they act as “mixing vessels” in which influenza viruses can reassort, mutate, adapt, and potentially acquire the ability to infect humans. Recently, H4 virus infection has been reported in pigs, indicating that this virus could be sustained among them by continuous viral infection and may accidentally be transmitted to other species. Although no human cases of H4 influenza have been reported so far, H4 viruses could represent a threat to humans in the future. The paper published in this Special Issue [6] reported the selection and development of an H4 inactivated influenza whole-particle vaccine prepared from an influenza virus isolated from wild birds. The vaccine is highly immunogenic in mice, elicits neutralizing antibodies with cross-reactivity, and could be an effective countermeasure in the event of a pandemic caused by the H4 influenza virus. 

A further interesting paper is provided by Tasker et al. [7], who aimed to evaluate the safety and immunogenicity of a single dose of an intranasal influenza vaccine candidate based on a replication-deficient adenovirus serotype 5 (Ad5) vector platform. The candidate vaccine proved to be well-tolerated and to elicit a broad immune response, including humoral, cellular, and mucosal immunity. Noteworthy, the antibody responses were maintained for at least one year post vaccination on average. 

Considering the contribution of cell-mediated immunity to protect against severe disease, even without an antibody response, Tan et al. [8] quantified the persistence of vaccine-related T cell epitopes in circulating swine influenza A strains sequenced between 2013 and 2017. They used the epitope content comparison tool, a useful method of comparing vaccines and circulating strains but also for identifying the efficacy of existing vaccines against emerging infections from a cellular immunity perspective, underlying the importance of vaccines based on highly conserved epitopes from circulating viruses.

The last two studies focused on LAIVs. The first one, by Caceres et al. [9], explored the possibility of incorporating the gene encoding the IgA-inducing protein (IGIP) in LAIVs against H1N1 influenza A virus in order to better stimulate protective antibody responses. The choice of IGIP modification is related to its potential as a natural vaccine adjuvant. The results are really promising, showing that natural adjuvants could improve the safety of LAIVs, maintaining and even improving upon the protective response against influenza A viruses. The final study [10] investigated the efficacy of LAIVs against influenza B, which can affect all age groups, though some studies have provided evidence that subjects ≤18 years old are more susceptible to infection [11]. In this study a vaccine candidate was generated by incorporating mutations E48K and K319E, along with attenuating mutations E580G and S660A, into the PB1 segment of the B/Brisbane/60/2008 strain with the aim of exploring the contribution of the E48K mutation to the stability of the K319E mutation. The results provided evidence of the compensatory effect of the E48K mutation to stabilize the K319E mutation, maintaining the safety and efficacy profile of LAIVs against influenza B viruses.

Altogether, the articles collected in this Special Issue provide an overview of different innovative approaches on the development of novel platforms, technologies, and next-generation vaccines against influenza viruses. The upcoming decades look very promising. The lessons learned during the COVID-19 pandemic has highlighted the importance of having different available technologies and approaches in the vaccine field to try to fight the sudden emergence of new viruses with pandemic potential.
